# Smartphone gaming induces dry eye symptoms and reduces blinking in school-aged children

**DOI:** 10.1038/s41433-022-02122-2

**Published:** 2022-06-06

**Authors:** Ngozi Charity Chidi-Egboka, Isabelle Jalbert, Blanka Golebiowski

**Affiliations:** School of Optometry and Vision Science, Faculty of Medicine and Health, UNSW Sydney, Sydney, NSW 2052 Australia

**Keywords:** Eye manifestations, Education

## Abstract

**Purpose:**

Smartphone use by children is rising rapidly, but its ocular surface impact is unknown. This study examined the effect of smartphone use on blinking, symptoms, and tear function in children.

**Methods:**

Prospective intervention study where 36 children aged 6–15years (14 M:22 F) played games on a smartphone continuously for one hour. Symptoms (SANDE, IOSS, NRS) and tear film (lipid layer thickness, tear secretion, stability) were assessed before and after gaming. Blink rate and interblink interval were measured in situ using an eye tracking headset, before (during conversation) and continuously throughout gaming. Symptoms and tear film changes were examined using paired *t*-tests. Changes in blinking throughout one hour were examined using repeated measures ANOVA, post-hoc comparisons with Bonferroni correction. Associations examined using Pearson bivariate correlation. Significance level was 0.05.

**Results:**

Symptoms worsened following one hour smartphone gaming (SANDE + 8.2units, *p* = 0.01; IOSS + 1.3units, *p* < 0.001; NRS-average +6.3units, *p* = 0.03; NRS-comfort +7.6units, *p* = 0.04; NRS-tiredness +10.1units, *p* = 0.01), but tear film remained unchanged. Blink rate reduced from 20.8 blinks/min to 8.9 blinks/min (*p* < 0.001) and interblink interval increased from 2.9 s to 8.7 s (*p* = 0.002) within the first minute of gaming relative to baseline conversation, and this effect remained unchanged throughout one hour of gaming.

**Conclusions:**

Smartphone use in children results in dry eye symptoms and immediate and sustained slowing of blinking, with no change in tear function evident up to one hour. Given the ubiquitous use of smartphones by children, future work should examine whether effects reported herein persist or get worse over a longer term causing cumulative damage to the ocular surface.

## Introduction

Digital device use among children has rapidly become ubiquitous [1]. Smartphones are the most commonly used digital devices [2–4]. More than 66% of United Kingdom children aged 5–16 years old and 83% of US children by age 15 years, own a smartphone [2, 4]. Similar ownership trends are observed globally among school-aged children, including in Australia, Europe and South-East Asia [5–7]. Children substantially exceed the 2 h per day of screen time recommended by the World Health Organisation [2] and by national health bodies [8–10] and this has been greatly exacerbated by the COVID-19 pandemic, in part due to increased isolation and at-home education [11, 12].

Excessive screen viewing during digital device use is associated with adverse general health and mental health outcomes in children [13, 14]. The adverse ocular effects of digital device use include progression of myopia in children and adolescents [15, 16]. The tear film and ocular surface may also be impacted [17, 18]. Evidence suggests that the odds of eye fatigue and strain related ocular symptoms in children and adolescents increase after more than 2 h of smartphone use [19]. The American Academy of Paediatrics recommends the 20-20-20 rule screen breaks (20 seconds break to look at an object 20 feet away every 20 min) for children as practiced by adults to avoid developing ocular symptom [20]. This recommendation for frequent screen breaks in children is viewed as important because children may not be as alert to symptoms of eye strain as adults [21].

Studies conducted in adults show that smartphone viewing adversely affects the ocular surface, causing discomfort, eye strain, sore eyes, and dry eyes, and altered tear film function and blinking [18, 22–25]. In adults, increased symptoms and other impacts on the ocular surface can occur with as little as one hour of smartphone use [18, 22–25]. Adverse effects on the ocular surface from prolonged and cumulative smartphone use have been reported only in children diagnosed with dry eye [17, 26–29]. However, an improvement in ocular surface symptoms and signs in children with dry eye after they had ceased using their smartphone for four weeks suggests that children may be similarly impacted as adults [17, 28]. The short-term or long-term effect of smartphone use on the ocular surface, including blinking, has not yet been prospectively investigated in children with healthy eyes.

Blinking is essential to ocular surface health and tear film homeostasis [30], and is a key marker altered during digital device use [18]. In adults, it is well established that blink rate is reduced with computer use [18, 31]. A reduction in blink rate has also been reported in young adults (university students) with one hour of smartphone gaming compared to baseline silence and listening to study procedure explanation by examiner [23]. Conversely, a trend to increase in blink rate and significant increase in incomplete blinks over one hour of smartphone reading although without comparing to any baseline activity has been reported in young adults [22]. In previous studies blink measurement was complicated, mostly requiring fixed head positions, and thus was challenging to measure during smartphone use. Therefore, a novel device which allows blink measurement without restricting head movement will be used in this study [32].

Smartphone use has become ubiquitous among children, yet the ocular surface impacts in this age group are not well understood. This study examined the effect of one hour of smartphone use on blinking, symptoms, and tear film indices in school-aged children.

## Methods

A prospective intervention study was conducted in adherence with the tenets of the Declaration of Helsinki and was approved by the UNSW Human Research Ethics Committee (approval number HC180420). Written informed consent was obtained from parents or guardians before enrolment in the study.

### Participants

Participants aged 6–15 years who understood English were recruited from the UNSW Sydney campus population and surrounding community. The upper and lower age limit of participants were determined and defined as previously reported, based on maturation of logical reasoning and ability to independently complete questionnaires [33, 34]. Minimum unaided or aided visual acuity of 0.2LogMAR at 6 m and 40 cm, and binocular vision (accommodation and convergence) normal for age were required [35]. Participants with any ocular conditions including eye allergies, contact lens wear within the last 24 h, any systemic conditions (e.g., Parkinson’s disease, diabetes) or medication use (e.g., menthol ointment, dopamine antagonists) likely to impact blinking were excluded [36, 37]. An estimated sample size of 45 participants was required to detect a mean difference of 3.6 blinks per minute in blink rate (primary outcome measure) with 80% power at a two-sided significance level of 0.05 (G*Power 3.1) and to account for a possible 20% attrition [38]. This sample size also allows detection of a mean difference of 5% for the eye symptom scores [17, 39], a mean difference of 2 s in non-invasive tear break-up time (NIBUT) and 0.02 mm in tear meniscus height (TMH) [22, 40, 41].

### Procedures

**Fig. 1:**
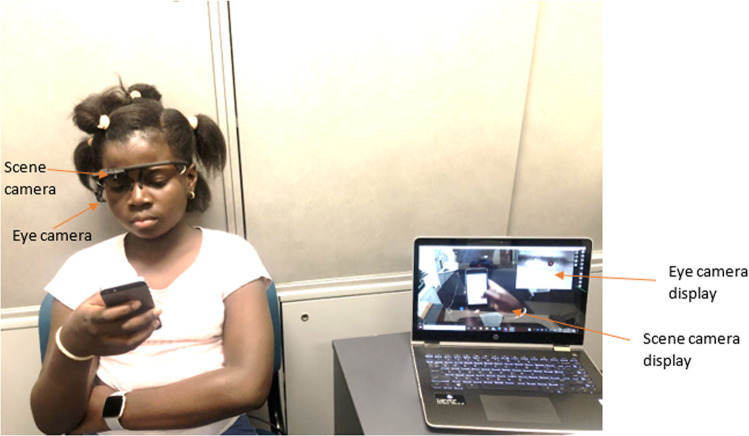
The experimental set-up showing the wearable eye tracking headset (Pupil Labs GmbH Berlin, Germany) during smartphone use. The eye camera display of the participant’s eye and the scene camera display enabling continuous monitoring of participant adherence by the examiner on the laptop. Child and parental consent were obtained for use of this image.

Using a smartphone (iPhone 5 s, Apple Inc. 2013), each participant played two games continuously for one hour, interchanging between Despicable Me: Minion Rush (Google Play Trailer; Gameloft, 2017) and Racing Penguin (Top Free Games, 2016). Participants were instructed to hold the smartphone at their habitual distance (Fig. 1) and were masked to the study purpose. The eye camera display (providing a view of the participant and their eye) together with the scene camera display (providing a view of what the participant is looking at) (Fig. 1) enabled continuous monitoring of participant adherence to the intervention in real-time and in the recording. The smartphone screen measured 4 inches (10.2 cm) diagonally, 1136 by 640 pixels for a resolution of 326 pixels per inch and was set to maximum brightness. Blinking was measured continuously; ocular surface symptoms and tear film function were assessed at baseline and after completing one hour of smartphone gaming. Study visits occurred between 12 noon and 6 pm each day to minimise possible diurnal variation [42] and the examination room was maintained at a temperature of 22 °C.

#### Ocular symptoms assessment

Participants self-completed three questionnaires which were selected based on feasibility of use in children [33] and responsiveness to change [34]: Instant Ocular Symptoms Survey (IOSS) [43], Symptoms Assessment in Dry Eye (SANDE) [44] and Numerical Rating Scale (NRS) [39]. Questionnaires were administered and scored as previously described [32, 33, 43, 44].

#### Tear film function assessment

Tear film measurements were conducted in the right eye only, in the following order: tear film lipid layer thickness (LLT) was assessed using LipiView® interferometer (Tear Science, Morrisville, NC), tear meniscus height (TMH) and non-invasive tear break-up time (NIBUT) were measured using Oculus® Keratograph 5 (Oculus®, Arlington, WA). LLT thickness was analysed based on the mean interferometry colour units (ICU) index automatically recorded by the LipiView® instrument [45]. TMH was measured at three locations to account for variability in TMH along the length of the lower meniscus: vertically below the pupil centre and directly below the nasal and temporal corneal limbal edge [46]. The average of the three measurements was recorded. The first detected tear break-up was automatically recorded for NIBUT [47].

#### Blink parameters assessment

In situ blink assessment was conducted as previously described [32] with a child-sized monocular (right eye) wearable eye tracking headset (Pupil Labs GmbH Berlin, Germany) (Fig. 1). The wearable eye tracking headset was worn over glasses for participants with habitual spectacle correction. Blink activity was measured continuously for 10 min during conversation (i.e., baseline) [32] prior to smartphone use and throughout one hour of smartphone gaming. To allow for adjustment and adaptation to wearing of the headset [48], data from the first three minutes of recording was discarded. Blinks were identified using open-source eye tracking software (Pupil Labs Core with Pupil software v2.0, Pupil Labs GmbH Berlin, Germany), based on visibility of the pupil [49, 50]. The Pupil software assigns a quality measure for the detected pupil in each video frame, referred to as “pupil confidence”. The pupil confidence value indicates how accurately the edge of the detected pupil fits an ellipse (range: 0 (no fit) to 1 (good fit) [32, 49]. Blinks are assumed to occur during pupil confidence drops evident when the pupil is obscured, hence high pupil confidence values in a recording are attributed to none obscured pupil, whereas low pupil confidence are relatively due to blinks detection [32, 49]. Poor pupil confidence unrelated to blinks can occur due to causes such as extreme gaze angles or pupil cover by eyelashes [49]. Blink data were included where more than 60% of pupil confidence (before blinking) values were above 0.6, as per the manufacturer’s recommendations for precise gaze detection [49]. The Pupil software blink detection algorithm identifies the onset of a blink (start of blink) when pupil confidence drops below the onset threshold for pupil confidence within the threshold time window. The offset of a blink (end of blink) is identified when pupil confidence recovers to above the offset threshold [49, 50]. Pupil confidence onset, offset and time window threshold values for this study were set at the manufacturer’s default values of 0.5, 0.5 and 0.2 seconds respectively [49]. Blink rate (number of blinks per minute) and interblink interval (the time between the end of one blink to the start of the following blink) data were obtained using the Pupil software Player module as described earlier [32]. The average blink rate and interblink interval were determined using the Pupil timestamps as earlier described [32], starting from baseline (data for last seven minutes), then first 10 min (0 to 10 min) of smartphone gaming and in blocks of 10 min throughout one hour recording.

### Statistical analysis

Statistical analysis was performed using IBM SPSS Statistics (version 26, 2019; Armonk, NY, USA). Data were tested for normality using Kolmogorov–Smirnov test (*p* > 0.05) and descriptive statistics, histograms, and QQ-plots. Differences in symptoms and tear film between baseline and after one hour were examined using paired t-tests. Changes in blink rate and interblink interval during first 10 min of smartphone gaming and throughout one hour gaming in blocks of 10 min compared to baseline were examined using repeated measures ANOVA and post hoc comparisons with Bonferroni correction. Associations between changes in blink parameters, ocular symptoms and tear film function were examined using Pearson bivariate correlation. All tests were two-tailed, and significance was established at *p* < 0.05.

## Results

Data from 36 participants (14 male:22 female) was analysed with mean age 10.3 ± 2.6 years (range 6–15 years). Data for nine of 45 participants were excluded in all analysis; including three participants (spectacle wearers) with pupil detection confidence below 0.6, and six participants whose recordings were not retrievable. The data loss to poor pupil detection confidence is most likely due to pupil obscuration by eyelashes as the participants exhibited no extreme gaze angles evident by the continuous eye monitoring during data collection. The nine excluded participants did not report severe eye symptoms or signs at either visit and their values were within the range of other participants. Participant demographics and baseline values for blink parameters, dry eye symptoms and tear film function are reported elsewhere and align with previously reported values for healthy children [32].

### Ocular symptoms

**Fig. 2:**
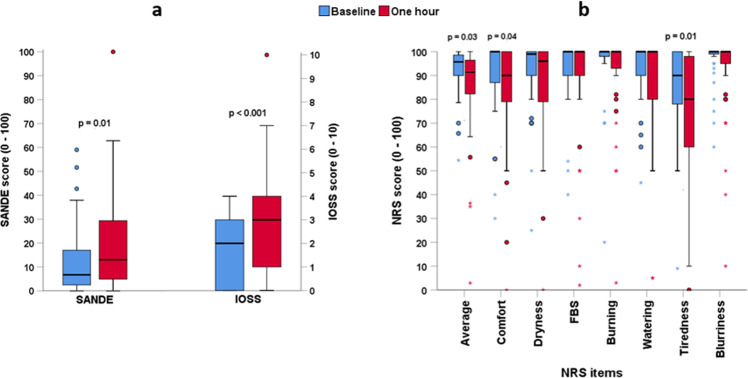
Ocular symptoms measured using Symptoms Assessment in Dry Eye (SANDE), Instant Ocular Symptoms Survey (IOSS) and Numerical Rating Scale (NRS). Ocular symptoms scores (median and interquartile range) at baseline (blue) and after one hour of smartphone gaming (red) for 36 school-aged participants with healthy eyes: **a**) SANDE and IOSS, **b**) NRS. Higher scores for symptoms indicate worse comfort, other than NRS where a higher score indicates better comfort. FBS denotes foreign body sensation. Blue and red circles represent mild outliers (symptom scores >1.5 to 3 times the interquartile range), blue and red stars represent extreme outliers (symptom scores >3 times the interquartile range).

Ocular symptoms measured using SANDE, IOSS and NRS (NRS comfort, NRS tiredness, and the average of all NRS symptoms) were significantly worse following one hour of smartphone gaming (Fig. 2).

### Tear film function

Tear film function (LLT, TMH and NIBUT) was not significantly impacted by one hour of smartphone gaming (Table 1).

**Table 1:** Tear film function measured using LipiView® interferometer (Tear Science, Morrisville, NC) and Oculus® Keratograph 5 (Oculus®, Arlington, WA), at baseline and after one hour of smartphone gaming in 36 school-aged participants with healthy eyes.

Tear film function	Baseline	One hour	*p* value
Lipid layer thickness^a^ (nm)	52.6 ± 16.3 (24–80)	56.7 ± 21.4 (22–100)	0.23
Tear meniscus height (mm)	0.26 ± 0.07 (0.15–0.45)	0.25 ± 0.06 (0.10–0.40)	0.25
Non-invasive tear break-up time (s)	10.4 ± 5.5 (2.7–23.8)	11.7 ± 7.6 (0.9–24.1)	0.36

Data are presented as mean ± SD (range).

^a^32 participants only are included for lipid layer thickness as values for four participants were above the upper cut-off of 100 interferometric colour units (ICU).

### Blink parameters

**Fig. 3:**
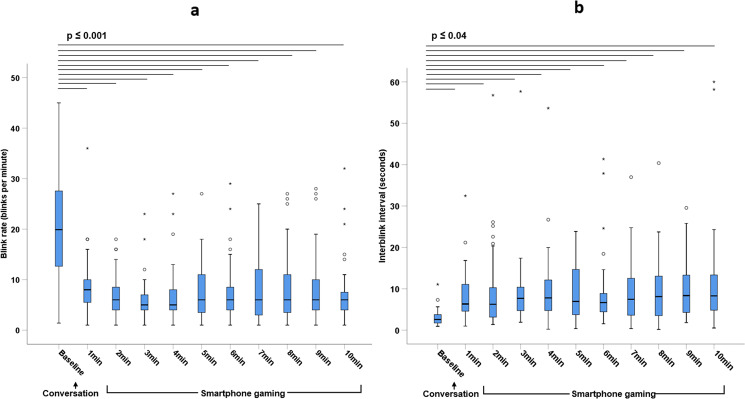
Blink parameters measured using an eye tracker headset at baseline during conversation and during the first 10 minutes of smartphone gaming. **a**) Blink rate and **b**) Interblink interval (median and interquartile range) in 36 school-aged participants with healthy eyes. Significant differences between timepoints are represented by horizontal lines. Circles represent mild outliers (measurements >1.5 to 3 times the interquartile range) and stars represent extreme outliers (measurements >3 times the interquartile range).

**Fig. 4:**
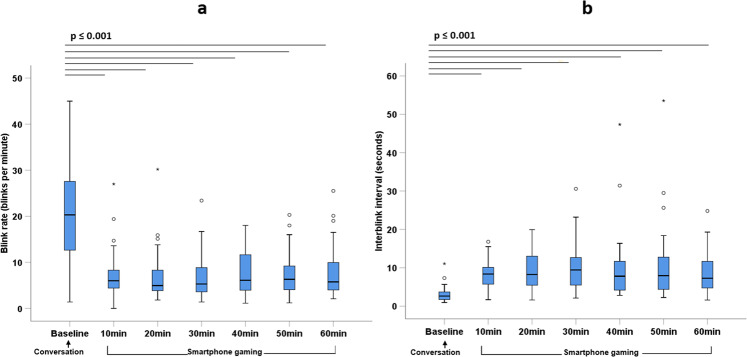
Blink parameters measured using an eye tracker headset at baseline during conversation and during one hour of smartphone gaming. **a**) Blink rate and **b**) Interblink interval (median and interquartile range) in 36 school-aged participants with healthy eyes. Significant differences between timepoints are represented by horizontal lines. Circles represent mild outliers (measurements >1.5 to 3 times the interquartile range) and stars represent extreme outliers (measurements >3 times the interquartile range).

Blink rate reduced (*p* ≤ 0.001) and interblink interval increased (*p* ≤ 0.04) in the first minute of smartphone gaming relative to the baseline values measured during conversation (Fig. 3). Thereafter, blink parameters remained unchanged throughout one hour of gaming (*p* ≥ 0.1) (Figs. 3 and 4).

### Associations between changes in blink parameters and symptoms

The worsening in ocular symptoms between baseline and one hour of smartphone gaming was not associated with the decrease in blink rate or increase in interblink interval over the same time (Supplementary data Table 1).

## Discussion

This is the first intervention study to examine the effects of smartphone use on the ocular surface and blinking in children. One hour of smartphone gaming led to increased symptoms of dryness, discomfort, and tiredness in children but did not impact tear film function. Blink rate decreased and interblink interval increased within the first minute of gaming on a smartphone, and this effect was maintained throughout one hour of gaming.

This is the first report of increased symptoms with smartphone use in children with healthy eyes. Elevated symptoms following one hour of smartphone use are consistent with findings in adults [18, 22–24]. Use of smartphones and tablets for 12 min to 4 h reading or gaming induces symptoms of ocular discomfort in adults with healthy eyes [22, 24, 25, 51, 52]. Investigations of ocular surface effects of smartphone use in children have focussed on children with dry eye. Studies in school-aged children with dry eye found increased dry eye symptoms with self-reported smartphone use of more than one [27] and 3 h [29] per day, relative to control with healthy eyes. Similarly, studies conducted in school-aged children with dry eye showed a reduction in ocular symptoms following cessation of smartphone use for one month [17, 28]. The symptom of tiredness and discomfort found in this study aligns with previous result in adults [22, 25], suggesting there is an important impact of smartphone use on visual fatigue [25].

This study showed for the first time that smartphone use rapidly reduces blink rate in children. Blink parameters have been sparsely investigated in children [29, 53]. A single study of school-aged children in Indonesia showed reduced blink rate with self-reported smartphone use of more than 3 h per day compared to control with less than 3 h of use [29]. In adults, one study found reduced blink rate after an hour smartphone gaming compared to baseline one minute of listening to examiner [23]. After as little as one minute of gaming, blink rate reduced rapidly to 8.9 blinks/min from a baseline of 20.8 blinks/min in this study. This aligns with reduced blink rate found after as little as 3 min of gaming on a computer in adults, measured relative to conversation [38] and undirected distance gazing [54, 55]. The findings from the present study taken together with the body of evidence summarized above suggest that blink rate is slower during tasks requiring greater concentration, (e.g., gaming) [18, 23, 31, 38, 56] compared to during less engaging moments [54, 57]. In agreement, evidence suggests similar reduction in blink rate during reading from printed text or digital device compared to baseline conditions [58, 59].

Interblink interval has not been previously reported in children with healthy eyes and it is sparsely reported in adults [18, 53]. The increased interblink interval during smartphone gaming found in this study contrasts with a study in adults which found reduced interblink interval with computer use [60]. However, this was linked to dryness from the exposed ocular surface area resulting in an increased blink reflex to maintain ocular surface homeostasis and comfort [60]. Interblink interval may be modulated by factors that directly affect ocular surface homoeostasis [60] and by the level of concentration required during a task [61]. For example, a study of school-aged children with dry eye found interblink interval increased from 2.89 seconds at first presentation to 4.58 seconds one month after they stopped smartphone use [28]. The differing study design and definition of interblink interval chosen by Dash et al in their study [28] which is at odds with the commonly used definition of time between the end of a blink and the start of another blink [32], makes it difficult to compare with the present study.

No further change in blink parameters occurred after the first minute of smartphone gaming, up to one hour. A similar intervention study in adults found a trend for a gradual increase in blink rate from 1 to 60 min of smartphone reading but did not compare to another task at baseline [22]. The same study [22] showed a significant rise in incomplete blinks during this time, while the rate of complete blinks was unchanged. In contrast, another study in adults reported a continuous slowing of blink rate during the first one to 40 min of smartphone gaming, with no change between 40 and 60 min [23]. Other evidence in adults suggest that change in blink rate during tasks is significant if measured continuously during task without any interruption [60, 62], thus it was essential to measure blink rate throughout task as in this study. Spontaneous blink rate is known to vary depending on type of activity or cognitive demand [57, 63, 64]. The lack of control group is a potential limitation of this study: it cannot be excluded that blink rates and dry eye symptoms could change simply by asking participants to sit in an indoor environment for one hour without any smartphone gaming. However, this is unlikely, as shown by the pre-intervention control measurement of 10 min of blinking during conversation while not engaged in the smartphone gaming task. These measurements are distinctly different to those during the gaming task. Notably, as smartphones may be used in many positions, the wearable eye tracking headset used in this study enabled precise blink capturing irrespective of the head or gaze position [32, 49].

No associations were found between changes in ocular surface symptoms before and after one hour of gaming on a smartphone, and changes in blink parameters over the same timeframe. It has been speculated that a reduced blink rate and/or extended interblink interval may disrupt ocular surface homeostasis, thus causing increased ocular surface discomfort [30, 65, 66], tear dysfunction and dry eye [42, 54, 60, 67]. Whereas some studies did not find direct associations between symptoms and blink rate during digital device use [59, 65, 68], others report that worsening of ocular symptoms was associated with a rise in incomplete blinks during reading on a smartphone for one hour [22] and a computer for 15–20 min [59, 68]. Complete blinking is essential to replenish tear film on the ocular surface and maintain ocular comfort [69]. Blink amplitude (complete and incomplete blinking) was not characterised in the current study. Analysis of blink amplitude during in situ blink measurement is an essential next step to better understand the role of blinking in smartphone associated discomfort.

One hour of smartphone gaming did not affect tear film function. This finding aligns with previous reports of no impact on tear film (lipid layer thickness, tear secretion, stability) in adults after one hour of gaming [24] or reading [22] on a smartphone. In contrast, reduced tear stability was reported in adults after one hour gaming on a tablet [25], and after 4 h gaming on a smartphone [24]. A single study in adults reported increased tear volume following one hour of smartphone gaming and movie viewing but the authors speculated this may have been a result of reflex tearing [23]. A study in school-aged children showed reduced tear stability and tear volume with a self-reported history of smartphone use of 3 h or more per day [29]. Improved tear stability was found in a group of school-aged children with dry eye one month after they stopped smartphone use [17]. Any effects on tear film of smartphone use may be transient [22], or evident only after extended use of up to 4 h [24]. Notably, measurements in the present study were conducted immediately (within five minutes after smartphone gaming was stopped), in order to detect any transient changes to tear film function. The lack of relationship between ocular surface symptoms and signs as found in this study nevertheless remains consistent with the bulk of the existing literature in adults and children [32, 70].

## Conclusions

This is the first study in children to examine ocular symptoms, blink parameters in situ, and tear film indices following smartphone use. One hour of smartphone gaming in school-aged children with healthy eyes quickly worsened ocular comfort, and rapidly slowed the blink rate to one third of that at baseline, with much longer open-eye periods between blinks. These effects were sustained throughout one hour. In the short term, changes in ocular symptoms and blinking were not accompanied by disturbances to the tear film.

Given the ubiquitous and rapidly rising use of smartphones by children globally, a better understanding of their ocular surface effects in this younger population will help to mitigate potential adverse impacts in the long term. Knowing that hours of smartphone use in the real world are longer than the short-term (one hour) intervention in the present study, it is reasonable to consider that the ocular symptoms and blink effects reported herein will persist or get worse over a longer term, causing cumulative damage to the ocular surface. Children may thus be at risk in the longer term of developing ocular surface disease and dry eye from excessive use of smartphones. This research demonstrates the rapid impact of screen viewing on eye health in children and these findings can help to inform recommendations for use of digital devices, including smartphones, by children. This work highlights blinking as a useful indicator of ocular surface changes in future investigations of the effects of prolonged and/or repeated use of smartphones and digital devices on the ocular surface of children.

## Summary

### What was known before

- Smartphones are the most commonly used digital devices.
- Children substantially exceed the World Health Organisation recommended screen time of 2 h per day.
- Excessive screen viewing during digital device use is associated with adverse general health and mental health outcomes in children.
- Digital device use causes adverse ocular effects such as myopia progression in children.
- Smartphone viewing adversely affects the ocular surface, causing discomfort, eye strain, sore eyes, and dry eyes, and altered tear film function and blinking in adults with healthy eyes.
- The effect of short-term, prolonged or cumulative smartphone use on the ocular surface, including blinking, has not yet been prospectively investigated in children with healthy eyes.

### What this study adds

- This is the first intervention study to examine the effects of smartphone use on the ocular surface and blinking in children.
- One hour smartphone use in school-aged children with healthy eyes quickly resulted in decreased ocular comfort, slowed the blink rate to one third, with much longer open eye periods between blinks.
- This study presents important findings that will be of interest beyond the field of ophthalmology.

## Supplementary information

Supplementary Table 1

## Data Availability

The datasets generated during and/or analysed during the current study are available in the Mendeley Data repository, https://data.mendeley.com/drafts/zn64p6r992.
