# Supplementary material for: Smartphone gaming induces dry eye symptoms and reduces blinking in school-aged children
Source: Eye (Lond). 2022 Jun 6;37(7):1342–9. doi: 10.1038/s41433-022-02122-2 (PMC9169596; doi:10.1038/s41433-022-02122-2)
Supplement: Supplementary file 1 — Supplementary Table 1 [file 41433_2022_2122_MOESM1_ESM.docx]

##### **Supplementary Data**

Supplementary **Table 1.** Associations between changes in spontaneous blink parameters (blink rate, interblink interval) and changes in ocular symptoms from baseline to after one hour smartphone gaming in 36 school-aged participants with healthy eyes.

*Abbreviations: IOSS, Instant Ocular Symptoms Survey; SANDE, Symptoms Assessment in Dry Eye; NRS, Numerical Rating Scale.*

| **Variables** | **Blink rate (blinks per minute)** | | **Interblink interval (seconds)** | |
| --- | --- | --- | --- | --- |
| ***Ocular Symptoms*** | ***r*** | **p-value** | ***r*** | **p-value** |
| IOSS | -0.09 | 0.61 | -0.06 | 0.75 |
| SANDE | -0.13 | 0.44 | 0.15 | 0.40 |
| SANDE Frequency | -0.12 | 0.49 | 0.10 | 0.56 |
| SANDE Severity | -0.16 | 0.34 | 0.08 | 0.66 |
| NRS Average score | 0.18 | 0.29 | -0.12 | 0.49 |
| NRS Comfort | 0.03 | 0.85 | -0.06 | 0.71 |
| NRS Dryness | 0.09 | 0.61 | 0.14 | 0.43 |
| NRS Foreign body sensation | -0.06 | 0.72 | -0.07 | 0.69 |
| NRS Burning | 0.31 | 0.06 | -0.001 | 0.99 |
| NRS Watering | 0.20 | 0.24 | -0.19 | 0.27 |
| NRS Tiredness | 0.31 | 0.07 | -0.27 | 0.12 |
| NRS Blurriness | 0.14 | 0.42 | -0.05 | 0.79 |

Note: Tear film function is not included in this analysis as no statistically significant change over time was noted in these variables.
